# Exploiting Linear Support Vector Machine for Correlation-Based High Dimensional Data Classification in Wireless Sensor Networks

**DOI:** 10.3390/s18092840

**Published:** 2018-08-28

**Authors:** Lawrence Mwenda Muriira, Zhiwei Zhao, Geyong Min

**Affiliations:** 1 College of Computer Science and Engineering, University of Electronic Science and Technology of China, Chengdu 611700, China; zzw@uestc.edu.cn; 2 College of Engineering, Mathematics and Physical Sciences, University of Exeter, Exeter EX4 4QF, UK; g.min@exeter.ac.uk

**Keywords:** linear support vector machine, linear kernel, correlation, high dimensional multi-category data classification, wireless sensor network

## Abstract

Linear Support Vector Machine (LSVM) has proven to be an effective approach for link classification in sensor networks. In this paper, we present a data-driven framework for reliable link classification that models Kernelized Linear Support Vector Machine (KLSVM) to produce stable and consistent results. KLSVM is a linear classifying technique that learns the “best” parameter settings. We investigated its application to model and capture two phenomena: High dimensional multi-category classification and Spatiotemporal data correlation in wireless sensor network (WSN). In addition, the technique also detects anomalies within the network. With the optimized selection of the linear kernel hyperparameters, the technique models high-dimensional data classification and the examined packet traces exhibit correlations between link features. Link features with Packet Reception Rate (PRR) greater than 50% show a high degree of negative correlation while the other sensor node observations show a moderate degree of positive correlation. The model gives a good visual intuition of the network behavior. The efficiency of the supervised learning technique is studied over real dataset obtained from a WSN testbed. To achieve that, we examined packet traces from the 802.15.4 network. The technique has a good performance on link quality estimation accuracy and a precise anomaly detection of sensor nodes within the network.

## 1. Introduction

The miniaturization of computing and sensing technologies enables the development of tiny, low-power, and inexpensive sensors, actuators, and controllers. These are sensing systems that typically interact closely with the physical world and are designed to perform a limited number of dedicated functions. Sensing is a technique used to gather information about a physical object or process, including the occurrence of events (for example, changes in state such as a drop in temperature or pressure). A device performing such a sensing task is called a sensor [1].

Sensors link the physical with the digital world by capturing and revealing real-world phenomena and converting these into a form that can be processed, stored, and acted upon [2]. Wireless Sensor networks (WSNs) are composed of cooperating sensor nodes that can perceive the environment to monitor physical phenomena and events of interest [3]. The dynamic environment makes the wireless communications vulnerable to distortion. The exposure of WSN to these external factors limits their performance due to their computing, storage and energy constraints.

The fast development of information from users and their applications; their networks and the cloud [4] have generated information diversification and complex relationship among information data [5]. Internet of Things that will connect the Internet with any kind of devices, living beings, and things will be the largest source of data in the near future. Wireless Sensor Networks are expected to have a key role in the realization of the future Internet of Things. Understanding the data deeply (the scale, structure, dimension, and correlation) guides the development of networks and information. Data correlation can solve the problems in data clustering, personal query and social network prediction [5,6].

Empirical WSN data correlations may exist in sensor network in three forms: correlation existing between sensor node observations on its history (temporal correlation), correlation existing between sensor node observation and neighboring nodes observation (spatial correlation), and correlation existing among attributes of sensor nodes (attribute correlation) [7]. Apart from the three basic types of data correlations mentioned, considering the temporal and spatial correlations together forms spatiotemporal correlations [8]. The combined correlation increases the detection rate of events on real dataset [9].

A major challenge for sensor nodes is sending sensed data to their sink in a reliable and energy efficient way, due to their limited resources in transmission, storage, computational memory and energy [10]. With the advancement in microelectromechanical systems (MEMS) technology, it is possible to deploy large scale WSNs, which introduce many data to be processed, transmitted and received. Transmitting all data back to a base station for processing and making inferences is impossible due to the sensor bandwidth constraints [11]. In addition, other challenges arise such as faulty node and outlier detection in target large-scale networks.

The utilization of machine learning seems to be one of the most convenient solutions for detecting anomaly in WSNs. Discovering an anomaly is a major principle to ensure a mundane functioning of WSNs [2]. Anomaly detection in WSNs is a consequential aspect of data analysis in order to identify data items which does not conform to an expected pattern in a dataset. It is paramount that the anomaly of sensor node is detected to obtain precise information to consequently make efficacious decisions by accumulated information.

For anomaly detection and utilization of data correlation for high dimensional classification in WSNs, there is a need for applying Support Vector Machine (SVM) with a kernel trick technique to mitigate such challenges. Some of the common types of kernel tricks applied are: Linear kernel, Polynomial Kernel, Radial Basic Function (RBF) kernel and Sigmoid Kernel [12]. These kernels are used to map dataset into high dimensionality feature space. In addition, the function of these kernels are to take dataset as input and transform it into the required form, with little computational cost even in very high-dimensional spaces. RBF kernel is the most popular kernel trick applied to non-linearly separable boundaries. Thus, the linear kernel works well if the datasets are linearly separable. However, the complexity of the RBF kernel grows with the size of the training. It is much easier to over-fit a complex model, because they have more hyperparameters to tune. This makes training of an RBF kernel SVM more computational costly and, furthermore, during prediction, the projection into “infinite” higher dimensional space where the data become linearly separable is more expensive than using LSVM [13,14].

**Figure 1:**
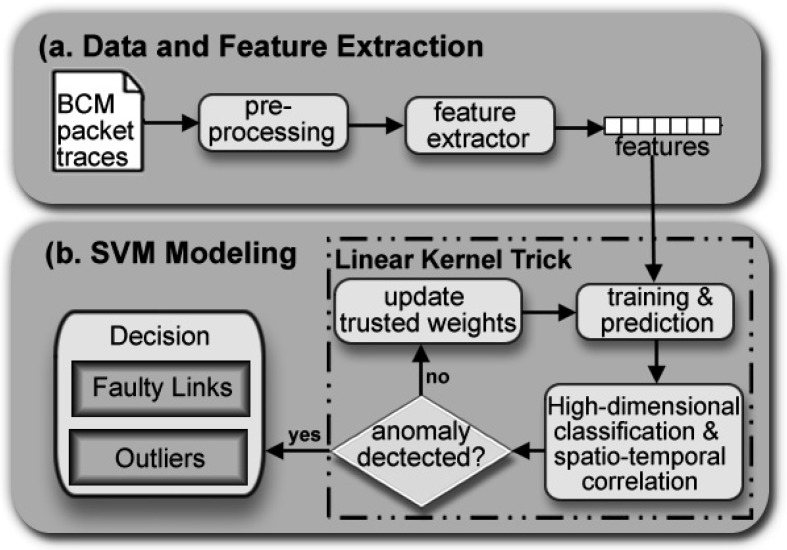
Kernelized Linear Support Vector Machine (KLSVM) Model: the Data-driven framework for reliable link classification.

To overcome this problem, we study a Kernelized Linear SVM technique for correlation-based high dimensional data classification and anomaly detection in WSNs. This approach is a linear classifying technique that learns the “best” parameter settings. The focus of our classification approach is not on generalization but on the accuracy of our classification for WSN’s link quality estimates, which are linearly separable. Our investigations show that the scattered plots of sensor node observations would tend to cluster around straight, non-horizontal lines and the sensed data are linearly correlated. This strategic technique and with a proposed data-driven framework for reliable link classification (see Figure 1) will provide insight into spatiotemporal correlations, high dimensional multi-category classification and anomaly detection, given an understanding of the distributive characteristic of WSNs.

Prior studies of WSNs have observed that links have a wide range of PRR which can vary significantly overtime [15]. To determine whether the network behaves similarly or dynamically, we measured our packet reception rates on a 4×5 TelosB testbed. We borrowed an approach from [16] and used beacon-based measurement (BCM) to capture received beacon messages receptions/losses in bitmap denoted as “1” for reception and “0” for loss. From the bitmaps, we computed PRR, retransmission count and Conditional Probability Delivery Function (CPDF). We used PRR to measure link quality estimates and the estimates were classified in a similar approach used by Shu et al. [17] as: Faulty Link with 0% PRR, Very Bad Link with PRR<10%, Bad Link with 10%≤PRR<45%, Intermediate Link with 45%≤PRR<75%, Good Link with 75%≤PRR<90% or Very Good Link with PRR≥90%.

The contributions of this paper are threefold. Firstly, we designed a data-driven framework for reliable link classification that models KLSVM to reveal two phenomena: high dimensional multi-category classification and spatiotemporal correlations in the sensor network. The design framework also detects anomalies caused by noises. Secondly, we studied the KLSVM model in the context of machine learning. We applied KLSVM technique to model high dimensional data classification, capture data correlation and detect anomalies. Finally, from empirical packet link traces collected from the WSN to our experimental evaluation, we show that the KLSVM technique modeled high dimensional multi-category classification over a real dataset. The approach captured temporal and spatial data correlation in 2D and 3D plots from the sensed data. We show KLSVM algorithm detects anomalies within the sensor network and models accordingly to more accurate diagnosis results. To the best of our knowledge, this is the first attempt to introduce KLSVM to experimentally model the two phenomena of spatiotemporal data correlation and high dimensional multi-category data classification based on link quality estimations in the field of WSNs. In addition, the technique detects anomalies within WSN in a single generic algorithm, paving the way to a sizable number of possible domains which can benefit from this technique.

This article provides in-depth insight into the KLSVM model and its application on a real dataset from a data classification and correlation perspective. The remainder of this paper is organized as follows. Section 2 is on related works. Section 3 is an in-depth description of the KLSVM model. Section 4 presents results and discussion of KLSVM. Finally, Section 5 concludes this paper.

## 2. Related Work

### 2.1. Preliminary

**Figure 2:**
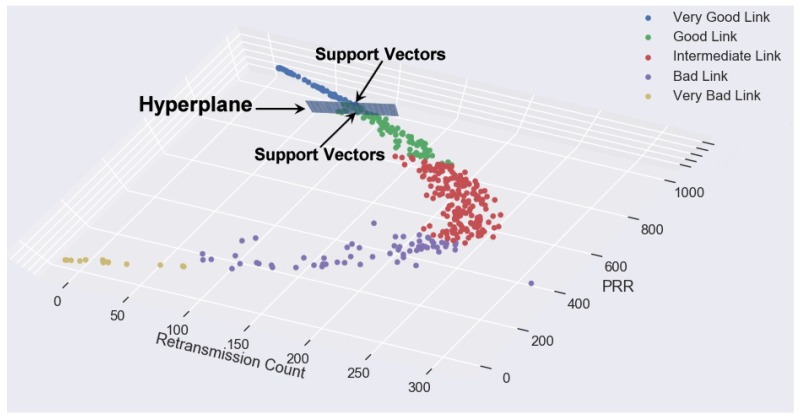
KLSVM—A correlation-based high-dimensional multi-category data classification model with a 3D hyperplane. The support vectors around the hyperplane are consequential training examples under SVM classification.

SVM algorithm is suited for extreme datasets separated by a Hyperplane. Thus, SVM can be defined as a frontier which best segregates two classes. If it does not have unoptimized decision boundary, it could result in greater misclassification on new data. Support vectors are defined as the data points that the margin pushes up against or points that are close to the opposing class. Therefore SVM algorithm implies that only these support vectors are essential, whereas other training examples are ignorable [18] as seen in Figure 2.

Let $x^{+}$ be the shortest distance to the closest positive point and $x^{-}$ be the shortest distance closest to the negative point, then we have a margin *M* of the separating hyperplane which is the distance between $x^{+}$ and $x^{-}$. The line (decision boundary) that segregates the two classes is referred as the Hyperplane. SVMs are used in multidimensional dataset and the data are referred to as vectors. In LSVM, classes are linearly separable but in those cases that the data are not linearly separable, a function is used to transform the data to high dimensional space [19]. The process is computationally expensive and kernel trick is used to reduce the computational costs [20]. Therefore, such a function that takes inputs as vectors in the original space and returns the dot product of the vectors in the feature space is called a Kernel Function also referred as Kernel Trick [12].

Using a kernel function, one can apply the dot product between two vectors that every point is mapped into a high dimensional space via some transformation, and using it to transform a non-linear space into a linear space. Aforementioned, some of the popular kernel types are as follows:

- Polynomial Kernel of degree *d* corresponds to a particular degree *d* expansion of the features $K\left(a,b\right)=\left(1+\sum_{j}a_{j}b_{j}\right)$. Data on the direction of a data point x>0, the larger the values of *x* shows a corresponding increase on the Gram Matrix $K(X,X^{\prime })$. Studies have shown polynomial kernels have been applied to overcome challenges in various fields, such as person re-identification problem by correctly matching person image from a set of gallery of person images in the field of machine learning [21], new malware detection in cyber-security [22], big data classification [23], facial recognition on multiple face images [24], human body movement and posture recognition in the medical field [25], facial emotion recognition [26] and classification of human brain images against mental health conditions in medical imaging [27].
- Radial Basic Function (RBF Kernel), also known as the Gaussian Similarity Kernel defined as $K\left(a,b\right)=\exp(-{\left(a-b\right)}^{2}/2\sigma^{2})$. It results in high values near the point of *x* and falling off as a Gaussian with some spread σ as it moves away from point *x*. Here, the parameter σ can be used to control over an under-fitting for σ chosen very large, all the data will look similar to any particular test point. This addition to the value *R* in Dual Optimization (discussed later in Section 3.6.2) which effectively controls the severeness of the penalty for misclassified points. RBF Kernel has been applied in different field of study such as detection of network intrusion in network security [28], enzyme discrimination in acidic and alkaline compound composition in biomedical engineering [29], emotion recognition from geometric facial features in digital image processing [30], stock market index predictions in the financial market [31], battery management system for optimized energy utility in electronic engineering [32], short-term wind power prediction in renewable energy [33], predicting traffic flow in smart city [34], load sharing and voltage compensation of micro-grids in electricity power distribution in electricity management [35] and data mining for regions susceptible landslide in China in the field of geographical information systems [36].
- Sigmoid Kernel $F\left(a,b\right)=tanh\left(ca^{T}b+h\right)$, also known as Hyperbolic Tangent Similarity Function, transits from zero and moves in the direction of the *x* solution, as long as that data is sufficient in the direction of *x*. However, far in the direction of greater values, *x* gets high similarity values [14]. Recent studies have shown the application of the following fields of research, such as protein sequence classification in Biomedical research [37], network anomalies Detection for intelligent power substation [38], landmark recognition in digital image processing [39] and facial expressions recognition for videos [40].

Choosing the correct kernel type is a non-trivial task and may depend on specific tasks at hand. No matter which kernel is chosen, one needs to turn the kernel parameter to get good performance of its classifier [41]. A popular parameter tuning technique includes K-Fold Cross validation [42]. K-Fold Cross-Validation is used to evaluate the performance of the machine learning algorithm. Cross-Validation gives the most mileage on the training data and performance metric are averaged across K-Fold [17].

Advantages of using SVMs include [43]: They are effective in high dimension spaces [18]; SVMs use the subset of training points in the decision function on support vector; it is memory efficient; different kernel functions can be specified for various decision functions; and kernel functions can be added together to achieve even more complex hyperplane. SVMs also have some drawbacks such as, if the number of features is greater than the number of samples, the algorithm is likely to give poor performance [44]. SVMs also do not directly provide probability estimates, these are calculated using expensive techniques such as the K-Fold Cross Validation.

SVM algorithm has numerous application for example: medical imaging, regressional modeling, image interpolation, medical classification tasks, financial industry time series prediction, financial analysis, application in neural networks for coding theory and practice, faulty diagnosis, page ranking and benchmarking, and objection recognition [45].

Recent studies have shown SVM is not only a classification technique but also an algorithm that can detect fault and anomalies [2], identify outliers [46], estimate and predict link quality [47,48], be used as an energy efficient routing method [43], and monitor and detect structural damage [42]. Trinh et al. [49] applied a data-driven hyperparameter optimization technique to detect anomaly in sensor networks using one-class support vector machines wth radial bias functions kernelized. Gui et al. [42] used a three optimization-algorithm grid search, partial swarm optimization and Gaussian kernel function parameters for damage detection on architectural structures. When the number of features are too large SVM performs poorly. Ghaddar and Naoum-Sawaya [44] proposed an approach based on iteratively adjusting a bound on the $l_{1}$-norm of the classifier vector to force the number of selected features to converge towards the desired maximum limit.

### 2.2. High-Dimensional Classification Techniques

Shu et al. [17] proposed a link quality estimation mechanism based on a multi-class classification SVM, modeled on two Kernel functions (radial basis and polynomial). Their model combined spatial correlation based data aggregation and opportunistic routing that achieved an improved network performance. Gholipour et al. [47] tackled the problem of network congestion and its improvement on WSN’s throughput by using a multi-classification obtained from SVMs. They used a genetic algorithm to tune parameter in their simulations. Effective estimation for link quality guarantees reliable data transmission. The model estimates the current link quality accurately with a relative small number of trained packets. Salberg [50] used a linear SVM classifier to detect and classify objects in remote sensing images. The model was used to classify accurately the desired classes such as non-seal, adult seal and puppy seal.

### 2.3. Correlation Techniques in WSN

Data correlation is characterized among sensor nodes especially under densely deployments, affecting network’s performance. Focus by researchers on data correlation in WSN has influenced designing of efficient routing protocols [51]. As more and more sensors are being deployed in the environment to provide sensing data to support various Internet of Things (IoT) applications, use of temporal and spatial correlations among different sensor data identifies the usefulness and correctness of each sensor data for different applications under IoT [52]. Utilization of temporal correlation of mobile sensing nodes on crowdsensing for fine-grained urban environmental monitoring has been achieved by fusing the sensory data with correlated environmental information [53]. According to Kumar and Kumar [54], their model built on Spatial and Temporal Data Correlation Algorithm for Data Aggregation overcame the challenge of data aggregation during flooding. With the dynamic characteristics of WSN, spatiotemporal data correlation approaches have been applied to data collection, aggregations, dissemination and network evaluation [9,55].

Similarly, studies have shown data correlation techniques can monitor detect fault and anomalies in WSN [7], predict link quality and energy efficient routing methods [51,54]. Densely deployed sensor nodes are prone to severe data redundancy due to the readings of neighboring nodes. High data redundancy leads to more energy consumption of the network. Large scale deployment causes congestion during transmission that leads to data losses and data retransmission is costly to WSN because of their limited energy budget [8]. Huang et al. [51] proposed a correlation aware opportunistic routing protocol that reduces redundancy by aggregating correlated data from selected forwarding nodes and the opportunistically forwarding the aggregated data to the sink. Here, we show how data correlation technique can detect faulty, anomalies and outliers within the WSN.

### 2.4. Anomaly Detection

Anomaly detection in networks and systems has enticed a large amount of attention recently. Erfani et al. [56] used a hybrid model to detect anomalies in large, high-dimensional datasets. Deep belief networks technique was applied for unsupervised training to extract features and while linear SVM was trained from the extracted features. However, Jedlinski and Jonak [57] used RBF-SVM for mechanical diagnosis to monitor vibration signal obtained from sensors on gearboxes. The Radial Basic Function was applied as the investigating method to enable early fault detection of gearboxes. An error-correcting output codes SVM is proposed for the multi-fault diagnosis of sensors. The author’s [58] approach solved the sensor fault feature extraction and an online identification problem on sensors. Garcia-Font et al. [59] also applied RBF for the work on study detecting anomalies in WSN for smart city. In clinical research, Wang et al. [60] used SVM with three independent classifiers to diagnose and classify information of healthy or pathological brain detection. The authors aimed to build an automatic classification system of brain images in magnetic resonance imaging (MRI).

## 3. The KLSVM Model

### 3.1. Linear Classifier

SVM is a classification technique that splits data in the best possible way between two regions. A hyperplane is used to best split the data by fitting the maximum margin between support vectors in the classified data. Support vectors are the data points for training on the margin. The process of maximizing the margin is a Constrained Optimization Problem which can be solved by using the Lagrange Multiplier technique [61].

The Lagrange’s Theorem states the following. Suppose *f* and *g* are functions of two variables that have continuous first partial derivative and ∇g≠0 throughout a region of the *xy-plane*. If *f* has an extremum $f(x^{\left(i\right)},y^{\left(i\right)})$ subject to the constraint g(x,y)=0, then there is a real number λ such that

(1) $$\nabla f\left(x^{\left(i\right)},y^{\left(i\right)}\right)=\lambda \nabla g\left(x^{\left(i\right)},y^{\left(i\right)}\right)$$

The number λ is called a Lagrange Multiplier.

LSVM is a common tool used for linear classifier which learns the “best” parameter setting. LSVM gives a stable decision boundary with the widest margin between support vectors. To explicitly maximize the decision boundary, it is important to optimize the margin classifier. Let $w_{i}$ be the weight associated with each feature $x_{i}$ and a constant term *b*, such that $b+w_{1}x_{1}+w_{2}x_{2}+\ldots$

### 3.2. Computing the Margin Width

**Figure 3:**
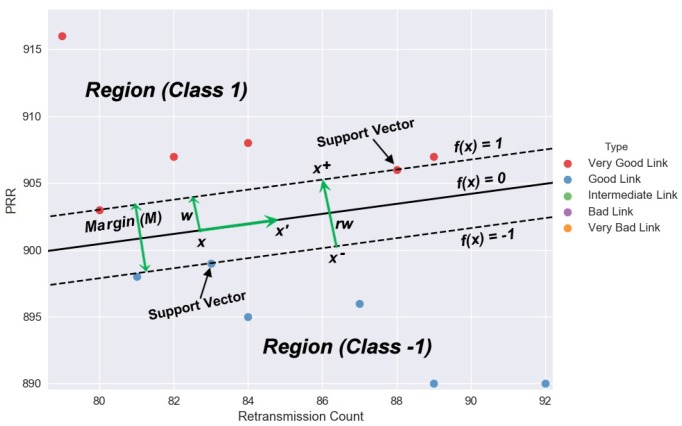
KLSVM: A modeled decision boundary obtained from the real dataset. The hyperplane separates two regions of the Very Good Link and the Good Link data points. The plot shows that the proposed method proves the accurate performance of the classifier.

To define the margin, let us assume that all parameters get all the link data correctly. For the setting of the parameter, the decision boundary is invariance to its scaling. Thus, we remove the scale invariance by defining class +1 in some region, class −1 in another and making those regions as far apart as possible (see Figure 3). Then, we can define this margin explicitly in terms of the hyperplane. Since no data are inside the hyperplane, we define the margin as the distance between the two regions:

(2a) $$f\left(x\right)=wx^{\prime }+b$$

(2b) f(x)>+1inclass+1region

(2c) f(x)<−1inclass−1region

all passing through zero centre

(2d) f(x)=0

We define margin $M=\left|\right|x^{+}-x^{-}\left|\right|=\left||rw|\right|$ and vectors $w\in {ℜ}^{n+1}$ to be perpendicular to the boundaries f(x)= −1, f(x)=+1 and f(x)=0 such that $wx+b=0\;\mathrm{and}\;wx^{\prime }+b=0\;\Rightarrow w(x^{\prime }-x)=0\;\mathrm{is}\;\mathrm{Orthogonal}.$

Choosing $x^{-}$ such that $f(x^{-})=-1$; let $x^{+}$ be the closest points with $f(x^{+})=+1$ defined as $x^{+}=x^{-}+rw$ since *w* is orthogonal to the planes. These closest points on the margin also satisfy $w\cdot x^{-}+b=-1$ and $w\cdot x^{+}+b=+1$. Since $x^{-}$ is on the negative hyperplane and $x^{+}$ is on the positive hyperplane, their linear responses are −1 and +1, respectively, such that

(3a) $$w\cdot (x^{-}+rw)+b=+1$$

(3b) $$\Rightarrow {r||w||}^{2}+w\cdot x^{-}+b=+1$$

(3c) $$\Rightarrow {r||w||}^{2}=1=+1$$

(3d) $$r=\frac{2}{{\left||w|\right|}^{2}}$$

Since

(4) $$M=\left||rw|\right|=\frac{2}{{\left||w|\right|}^{2}}\left||w|\right|=\frac{2}{\sqrt{w^{T}w}}$$

### 3.3. Maximum Margin Classifier

We optimize our parameter since it is a constrained optimization problem to best fit our margin. We do that by getting all the data points correctly as the maximized margin equation subject to the constraints. Thus, all our data lay in specified regions on the correct side of the margin, as shown in Figure 3.

(5) $$w^{*}=arg\max_{w}\frac{2}{\sqrt{w^{T}w}}$$

Finding the value of $w^{*}$ that maximizes ||rw||, we can equivalently find the value *w* that minimizes $w^{*}$ length.

#### 3.3.1. The Primal Problem

The vector $w_{j}^{2}$ with the smallest length will also be the $w^{*}$ with the largest inverse length. Similarly, minimizing squared length instead does not change the location of the minimizer, defined as

(6) $$w^{*}=arg\min_{w}\sum_{j}w_{j}^{2}\;(\mathrm{The}\;\mathrm{Primal}\;\mathrm{Problem})$$

subject to

$$y^{\left(i\right)}=+1\Rightarrow w\cdot x^{\left(i\right)}+b\geq +1$$

$$y^{\left(i\right)}=-1\Rightarrow w\cdot x^{\left(i\right)}+b\leq -1$$

by reforming the maximization as a minimization of the sum of $w_{j}^{2}$.

To enforce the data constraint, we have one constraint per data point. If $y^{\left(i\right)}=+1$, the linear response will be greater than +1. If $y^{\left(i\right)}=-1$, the linear response will be less than −1.

#### 3.3.2. Quadratic Program

The margin problem is an example of classic optimization problem called the Quadratic Program. We minimize the Quadratic Program of the parameters $\sum_{j}w_{j}^{2}$ in Equation (6) subject to a collection *m* linear constraint on the parameter at each data point that lies in the correct region. Framing this way makes easy to apply optimization algorithm designed for quadratic programs for reference, it is the formulation a Maximum Margin Classifier for Primal Problem.

It is convenient to compact these $w\cdot x^{\left(i\right)}+b\geq +1$ and $w\cdot x^{\left(i\right)}+b\leq -1$ constraints parameters to a single phrase that works for both positive and negative regions:

(7) $$y^{\left(i\right)}\left(w\cdot x^{\left(i\right)}+b\right)\geq +1$$

In Equation (7), $y^{\left(i\right)}$ is either +1 or −1. If it is *+1*, the linear response should also be positive and greater than one. Thus, the product $y^{\left(i\right)}\left(w\cdot x^{\left(i\right)}+b\right)$ will be the same. If y=−1, then the linear response should be negative and the product will be greater than one. Therefore, our linear response should be larger than one in magnitude.

### 3.4. Lagrangian Optimizer

Let f(θ) be our objective function and $g_{i}\left(\theta \right)\leq 0$ be our *Constraint Function*, where θ represent data *w* and *b* such that θ=(w,b) is defined as

(8a) $$f\left(\theta \right)=arg\min_{w,b}\sum_{j}w_{j}^{2}=w^{*}$$

subject to

(8b) $$g_{i}\left(\theta \right)=1-y^{\left(i\right)}\left(w\cdot x^{\left(i\right)}+b\right)\leq 0$$

by minimizing the weights subject to the constraint that enforces the correctness model of our margin on each data point.

Then, we introduce a Lagrange Multiplier λ used to enforce the constraint θ jointly with a simple constraint set

(9) $$\theta^{*}=arg\min_{\theta }\max_{\lambda \leq 0}f\left(\theta \right)+\sum_{i}\lambda_{i}g_{i}\left(\theta \right)$$

Introducing the Lagrange Multiplier λ for each constraint $g_{i}\left(\theta \right)$ is used to enforce the constraint. The *Lagrangian* is given by optimization over the original parameters $\min_{\theta }$ and $\max_{\lambda \leq 0}$, where θ is to be minimized while λ is to be maximized. There is also a simple constraint on $\max_{\lambda \leq 0}$ to be non-negative, so it is easy to initiate the values θ and λ together by gradient descent steps

(10a) $$g_{i}\left(\theta \right)\leq 0\;:\;\lambda_{i}=0$$

(10b) $$g_{i}\left(\theta \right)>0\;:\;\lambda_{i}\to +\infty$$

consider optimizing over $\lambda_{i}$ for any fixed data.

#### 3.4.1. KKT Complementary Slackness

If the constraint $g_{i}\left(\theta \right)$ is satisfied, then $g_{i}\left(\theta \right)$ is negative and the largest obtainable is zero by setting $\lambda_{i}=0$, If the constraint is not satisfied, $g_{i}\left(\theta \right)$ is positive, λ will increase, and then data will have to change to decrease $g_{i}\left(\theta \right)$ for that constraint. Any optimum to the original problem will be a saddle point of the new Lagrangian and vice versa.

(11) $$\lambda_{i}>0\Rightarrow g_{i}\left(\theta \right)=0$$

#### 3.4.2. Optimization of the Lagrange Multiplier

The *Lagrangian* can be optimized by enforcing inequality constraint, defined as

(12) $$w^{*}=arg\min_{w}\max_{\lambda \leq 0}\frac{1}{2}\sum_{j}w_{j}^{2}+\sum_{i}\lambda_{i}\left(1-y^{\left(i\right)}\left(w\cdot x^{\left(i\right)}+b\right)\right)$$

for θ>0 only on the margin.

Fixing λ can solve directly for *w* and *b* in terms of λ. This unconstrained quadratic function takes their derivates and sets them to zero. It gives the optimal $w^{*}$ to be a linear combination of the data, defined as

(13) $$w^{*}=\sum_{i}\lambda_{i}y^{\left(i\right)}x^{\left(i\right)}$$

and, since any support vector has y−wx+b,

(14) $$b=\frac{1}{Nsv}\sum_{i\in SV}\left(y^{\left(i\right)}-w\cdot x^{\left(i\right)}\right)$$

$\frac{1}{Nsv}$ averages the equation for numerical stability. Since $\lambda_{i}$ is zero for non-support vector points $y^{\left(i\right)}x^{\left(i\right)}$, the $\max_{\lambda \leq 0}$ boundary in Equation (12) and solution $w^{*}$ in Equation (13) depend only on the support vectors. Solve for *b* by plugging the margin of the hyperplane in its equation for the support vectors *sv*, by using solution $w^{*}$ to write solely in terms of θ as

(15) $$\max_{\lambda \leq 0}\sum_{i}\left[\lambda_{i}-\frac{1}{2}\sum_{j}\lambda_{i}\lambda_{j}y^{\left(i\right)}y^{\left(j\right)}\left(x^{\left(i\right)}\cdot x^{\left(j\right)}\right)\right]$$

subject to $\sum_{i}\lambda_{i}y^{\left(i\right)}=0$ since its a derivative with respect to b=0.

### 3.5. Dual Form

The optimal value *w* in terms of $\lambda_{i}$ is plugged in to get an optimization solely over λ. The resulting problem is regarded as the Lagrange Dual form of the original problem. Plugging in $w^{*}$ of Equation (12) and rearranging it, we find that the dual is given by $\lambda_{i}-\frac{1}{2}\sum_{j}\lambda_{i}\lambda_{j}y^{\left(i\right)}y^{\left(j\right)}\left(x^{\left(i\right)}\cdot x^{\left(j\right)}\right)$ of Equation (15) as a minimization over λ≤0. Enforcing stationary condition on *b*, the derivative with respect to b=0, since the original equation was linear with *b*, then $\sum_{i}\lambda_{i}y^{\left(i\right)}=0$ becomes a constraint. Notice Equation (15) is a quadratic function in λ with single linear constraint, so it is a quadratic program that optimizes *m* variables with 1 + *m* simple constraint of the objective function has $m^{2}$ dot products. The Lagrangian Dual is always lower bound on the original primal problem of the minimization over θ.

Quadratic problems such as these have a property called strong duality which states the value of this optimization over λ will be the same as the primal problem. This dual forms are used when *m* the number of data points is much smaller than the number of features *N*. Notice that in Equation (15) our optimization is now λ which is *m* evaluation of the objective of $\lambda_{i}-\frac{1}{2}\sum_{j}\lambda_{i}\lambda_{j}y^{\left(i\right)}y^{\left(j\right)}\left(x^{\left(i\right)}\cdot x^{\left(j\right)}\right)$ is then $\frac{1}{m^{2}}$ and optimizing is usually between quadratic and cubic *m* depending on the solver used, then optimization tolerance.

### 3.6. Linearly Non-Separable Data

For not linearly separable data, the margin constraints cannot be satisfied for any values of *w* and *b*; the large margin principle $\min_{w}\sum_{j}w_{j}^{2}$ for separable data suggests we should use a model with small parameters. However, if we are forced to have a non-zero error $\min_{w}\sum_{j}J\left(y^{\left(i\right)},w\cdot x^{\left(i\right)}+b\right)$, we should trade off.

#### 3.6.1. Slack Variable

**Figure 4:**
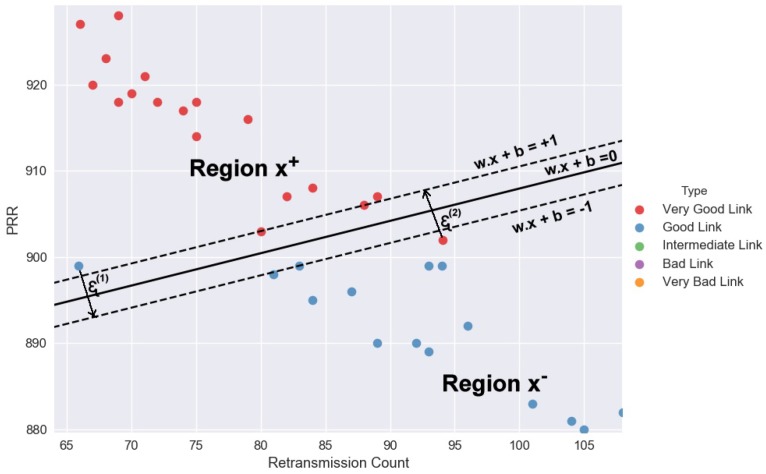
KLSVM: Linearly non-separable data points between the *Very Good Link* and the *Good Link* regions. The retransmission count patterns of these data points are inconsistent with the other data points of their classes.

The error that results from our prediction is caused by some of our solutions allowing some of our data points to violate the margin constraint. Thus, a Soft Margin is assigned a cost, for example, the distance by which they violated the constraints scale by a factor *R*. If the factor *R* is chosen to be very large, it pays a lot of attention to make sure no data violate the constraints margin if possible. On the other hand, if *R* is very small, maximize the margin for most data to allow some of them to violate it. Figure 4 shows we do so by adding the so called Slack Variables $\epsilon^{\left(i\right)}$ one for each data points, defined as

(16) $$w^{*}=arg\min_{w,\epsilon }\sum_{j}w_{j}^{2}+R\sum_{i}\epsilon^{\left(i\right)}$$

subject to $y^{\left(i\right)}\left(w^{T}x^{\left(i\right)}+b\right)\geq +1-\epsilon^{\left(i\right)}$ violating margin by $\epsilon^{\left(i\right)}\geq 0$.

$\epsilon^{\left(i\right)}$ measures the amount of data point *i* violating the margin constraint. $\epsilon^{\left(i\right)}$ is always non-negative or zero if the constraints are satisfied and adds a penalty to epsilon to our objective function balancing the margin in terms of margin squared $w_{j}^{2}$ with the amount of slack $R\sum_{i}\epsilon^{\left(i\right)}$. Notice the formulation of $w^{*}$ in Equation (16) remains a quadratic program; its quadratic objective in *w* and ϵ subject to linear constraint $y^{\left(i\right)}\left(w^{T}x^{\left(i\right)}+b\right)\geq +1-\epsilon^{\left(i\right)}$. For any weight *w*, we can choose ϵ to satisfy constraints by writing $\epsilon^{*}$ as a function *J* and optimizing directly. For any weight vector *w*, we can always choose a value ϵ to minimize its term and satisfy constraints. It means, first, we can always initiate a solution *w*, *ϵ* and *b* to something that satisfies the constraints even if it does not minimize the objective. Secondly, the optimal value ϵ given *w* is easy to select if data point *i* is not satisfied, the margin with $\epsilon^{\left(i\right)}=0$, then the smallest value ϵ, enforces these inequalities to be true and will accept two sides to be equal.

By choosing an optimal value for a given *w*, we can then optimize the resulting cost as function of *J* directly as

(17) $$J_{i}=\max\left[0,1-y^{\left(i\right)}\left(w\cdot x^{\left(i\right)}+b\right)\right]$$

where $\epsilon^{*}$ written as a function of *J* can be optimized directly to

(18) $$w^{*}=arg\min_{w}\sum_{j}w_{j}^{2}+R\sum_{i}J_{i}\left(y^{\left(i\right)},w\cdot x^{\left(i\right)}+b\right)$$

We find that the cost of *J* is only non-zero for data points that do not satisfy the margin constraint and for those points equal linearly with their distance to the margin.

#### 3.6.2. Standard Linear Classifier Optimization

For positive data point w·x+b→+1, the linear response is already greater than +1 and has no cost J=0. On the other hand, if it is less than +1, the cost will increase linearly with its distance from the margin. This kind of loss is called Hinge Loss. Its analytical form $J_{i}$ as shown in Equation (17) is piecewise linear, and is either a zero or positive course that increases away from the margin. Our overall optimization in Equation (18) is then a margin term $\sum_{j}w_{j}^{2}$ plus our stack variable *R* times the Hinge Loss $J_{j}$ and a balance between the margin term and stack variables, also defined as

(19) $$w^{*}=arg\min_{w}\frac{1}{R}\sum_{j}w_{j}^{2}+\sum_{i}J_{i}\left(y^{\left(i\right)},w\cdot x^{\left(i\right)}+b\right)$$

Dividing Equation (19) by *R*, we get that the optimal parameter minimizes the sum of the data term and hinge loss, that is, $\frac{1}{R}$ times an $l_{2}$ regularization terms of their weights. This equation forms as a standard linear classifier optimization, but the difference is the Hinge Loss and not regularizing the coefficient *b*.

We can optimize $\sum_{j}J_{i}\left(y^{\left(i\right)},w\cdot x^{\left(i\right)}+b\right)$ in whatever manner we like such as any standard Stochastic Gradient algorithm from the linear classifier. If we take the dual of the soft margin quadratic program, we obtain a quadratic program similar to before over only the Lagrange multiplier λ.

(20) $$\max_{0\leq \lambda \leq R}\sum_{i}\lambda_{i}-\frac{1}{2}\sum \lambda_{i}\lambda_{j}y^{\left(i\right)}y^{\left(j\right)}\left(x^{\left(i\right)}\cdot x^{\left(j\right)}\right)$$

subject to $\sum_{i}\lambda_{i}y^{\left(i\right)}=0$. The $\lambda_{i}$ is bounded from above as well by *R*. Intuitively, the equation says that, if the data point violates the margin constraints, the margin will penalize the Lagrange multiplier $\lambda_{i}$ of that data point. Then λ will increase until it is at most *R* times the violated distance, defined as

(21) $$w^{*}=\sum_{i}\lambda_{i}y^{\left(i\right)}x^{\left(i\right)}\;\forall \lambda \in \left(0,R\right)$$

Complimentary slackness tells that λ is non-zero only on data that are at the margin or on the wrong side, for positive data at any point where the linear response is less than equals to +1.

### 3.7. Gram Matrix

The dual form can be important when there are many features than data points, that is, N>>m, a key thing to notice about dual form of the SVM is that quadratic program involves the features *x* only through their dot products. In other words, the coefficient of *i* and *j* interaction of the term between $\lambda_{i}$ and $\lambda_{j}$ is the inner product of the data point of $x^{\left(i\right)}\cdot x^{\left(j\right)}$.

Let’s call this inner product $K_{ij}$, that is, $i^{th}j^{th}$ entry in the matrix *K* sometimes called the Gram Matrix. We can think of the quantity as measuring the similarity of two data points $x^{\left(i\right)}$ and $x^{\left(j\right)}$ through their dot product if and only if the vectors are in the same direction. It takes its maximum value and its orthogonal at zero; if they are on opposite directions, it has negative values.

### 3.8. Prediction

Interestingly, predictions also only involve dot products using our solutions $w^{*}$. We find that our prediction $\hat{y}$ for a new test point *x* is a linear combination of the dot products of *x* with the support vectors $x^{\left(i\right)}$ the points where $\lambda_{i}$ is non-zero, defined as

(22) $$\hat{y}=w^{*}\cdot x+b=\sum_{i}\lambda_{i}y^{\left(i\right)}\cdot x+b$$

Evaluating *b* is a bit more complicated but it is before any support vector with slack on λ, not equal to 0 or R will have a tight margin constraint that can be used to solve for *b*. Typically, *b* is kept updated when λ has been solved.

### 3.9. Kernel Function

In practice, linear functions may not correspond to any particular feature transform but often seems to perform well empirically. For extremely large data, however, kernels and SVMs are less common and LSVM with explicit features are more typical. For datasets $0(m^{2})$ cost, working in dual form may be too much and Primal optimizing the Hange Loss directly using Stochastic Gradient Descent is preferred [12].

#### 3.9.1. Kernelizing Linear SVM

LSVM has a simplified perceptron of a linear weight *w* on the input features resulting in a linear decision boundary f(x)=0 developed by a Lagrangian optimization in the form:

(23) $$\max_{0\leq \lambda \leq R}\sum_{i}\lambda_{i}-\frac{1}{2}\sum_{j}\lambda_{i}\lambda_{j}y^{\left(i\right)}y^{\left(j\right)}\left(x^{\left(i\right)}\cdot x^{\left(j\right)}\right)$$

subject to $\sum_{i}\lambda_{i}y^{\left(i\right)}=0$. That leads to an equivalent dual formation process in which the objective function depends on the matrix $K_{ij}=x^{\left(i\right)}\cdot x\left(j\right)$ on pairwise dot product called the Gram Matrix.

**Figure 5:**
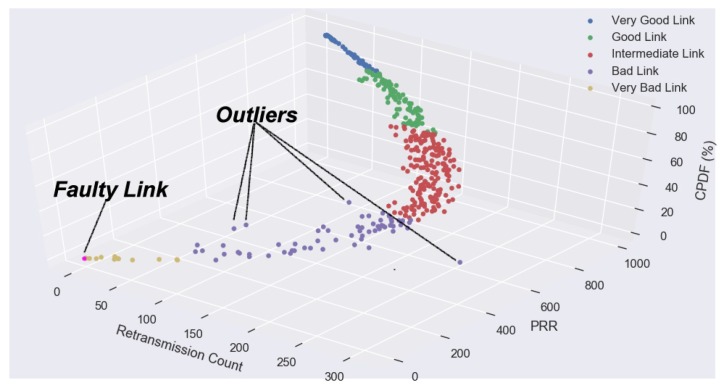
KLSVM: A modeled correlation-based high dimensional multi-category classification. In addition, the results show one detected failed link and four captured outliers, with extremely inconsistent behavior.

If our data are not linearly separable on the original feature $x_{1}$, we can add quadratic deterministic features, for example, moving from one feature $x_{1}$ to two features $x_{2}=\left(x_{1}^{2}\right)$ so that the data lie on a curve. In our new higher dimension space (see Figure 5), our data are more likely to be linearly separable. This affects the dual form of the sum using a feature transform Phi(x) $\hat{y}\left(x\right)=sign\left[w\cdot \Phi \left(x\right)+b\right]$. We transform $x^{\left(i\right)}$ and $x^{\left(j\right)}$ to find the new feature vectors, and then the dual form involves the dot products between these transformed vectors, defined as

(24) $$\max_{0\leq \lambda \leq R}\sum_{i}\lambda_{i}-\frac{1}{2}\sum_{j}\lambda_{i}\lambda_{j}y^{\left(i\right)}y^{\left(j\right)}\Phi \left(x^{\left(i\right)}\right)\Phi {\left(x^{\left(j\right)}\right)}^{T}$$

subject to $\sum_{i}\lambda_{i}y^{\left(i\right)}=0$.

Let us consider the polynomial features defined as

(25a) $$\Phi \left(x\right)=\left(1\sqrt{2x_{1}},\sqrt{2x_{2}}\ldots x_{1}^{2}x_{2}^{2}\ldots \sqrt{2x_{1}x_{2}}\sqrt{2x_{1}x_{3}}\ldots \right)$$

For the dual form, we need to compute inner product of two expanded feature vectors $\Phi \left(x^{\left(i\right)}\right)\Phi {\left(x^{\left(j\right)}\right)}^{T}$ by denoting $x^{\left(i\right)}$ and $x^{\left(j\right)}$ to *a* and *b* respectively. Listing our features for both points, we compute them as

(25b) $$\Phi \left(a\right)=\left(1\sqrt{2a_{1}}\sqrt{2a_{2}}\ldots a_{1}^{2}a_{2}^{2}\ldots \sqrt{2a_{1}a_{2}}\sqrt{2a_{1}a_{3}}\ldots \right)$$

(25c) $$\Phi \left(b\right)=\left(1\sqrt{2b_{1}}\sqrt{2b_{2}}\ldots b_{1}^{2}b_{2}^{2}\ldots \sqrt{2b_{1}b_{2}}\sqrt{2b_{1}b_{3}}\ldots \right)$$

We find that the dot product is the sum, defined as

(25d) $$\Phi {\left(a\right)}^{T}\Phi \left(b\right)=1+\sum_{j}2a_{j}b_{j}+\sum_{j}a_{j}^{2}b_{j}^{2}+\sum_{j}\sum_{k>j}2a_{j}a_{k}b_{j}b_{k}+\ldots$$

If we manipulate the sum algebraically, we find that it is equivalent to a much simpler computation

(25e) $$\Phi {\left(a\right)}^{T}\Phi \left(b\right)={\left(1+\sum_{j}a_{j}b_{j}\right)}^{2}=K\left(a,b\right)$$

denoting the value K(a,b) instead of actually linking our higher dimensional feature transform and then computing the inner product of those vectors, we could instead take the very simple non-linear function similarity (the kernel) and compute on the linear and the original number of features our expansion fee has; for example, quadratic features Φ(a) and Φ(b) have all squared features and computing their dot product in only 0(n) computations.

#### 3.9.2. Mercer’s Kernel

In addition, a non-linear function $K(X,X^{\prime })$ satisfying a particular condition called Mercer’s Condition can be viewed as corresponding to the dot product between transform vectors Φ(x) for some transformational fee:

(26) $$\int_{a}\int_{b}K\left(a,b\right)g\left(a\right)g\left(b\right)dadb>0$$

Then, the similarly function K(a,b)=Φ(a)·Φ(b) is also referred to as Mercer’s Kernel (condition). As a side note, this Mercer’s condition is effectively condition on the Gram Matrix K as a positive definite structure for any possible dataset x, defined as $g^{T}\cdot K\cdot g\geq 0$.

For polynomial features, there is direct response between a particular *K* and a particular feature vector Φ, however for arbitrary similarity function *K*, it might be quite hard to find exactly how vector feature Φ correspond to that *K*. In fact, many useful kernel functions correspond to infinite dimensions of Phi vectors. Thus, using such a kernel is equivalent to a linear classifier with an infinite number of constructed feature yet it is not computational harder as long as *K* itself is easy to compute. Then, we can calculate the gram matrix $K(X,X^{\prime })$ in 0 (m${}^{2}$) time in space and solve the resulting quadratic programming to quadratic cubic times.

## 4. Results and Discussion

### 4.1. Data Description

**Figure 6:**
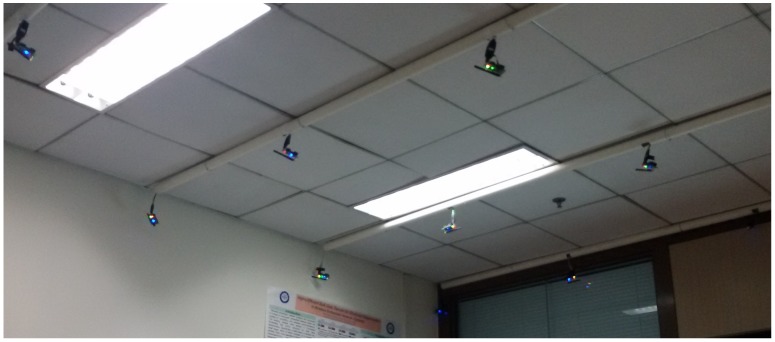
NGMI’s ceiling: Part of the 4 × 5 WSN testbed.

We considered a dataset gathered from a WSN deployment at the New Generation Mobile Internet Research Laboratory (NGMI) in the University of Electronic Science and Technology of China. The results are from a 20-sensor-node testbed on the laboratory’s ceiling. Figure 6 shows the sensor deployment in the laboratory. The nodes have identification numbers, *Node0* as the root node and the other nodes follow a sequential order (*Node1, Node2,..., Node20*). The nodes in this experiment run on TinyOS and use TelosB sensor motes.

We applied the BCM approach that uses the beacon receptions to provide historical statistics. To determine the actual packet traces results, two tunable modeling parameters were considered: window size *W*, representing the number of packets used in each single input, and the interval-packet interval *I*, representing the time interval between packets. The window size corresponds to the amount of historical information required by our experiment to estimate the link quality in our prediction. We programmed nodes on the meshed network topology testbed to broadcast 400,000 packets with an inter-packet interval of 50 ms using 16 channels. Each node transmitting 1000 packets to a relaying node or to the root node. Timing is an important factor for beaconed packet distribution and we needed a way that could concisely describe link behavior from the packet traces. At *I* = 50 ms, the WSN’s links stabilized to a near-perfect quality for our experiment and consequently our data collection technique also converged to a stable state. To measure the link features, we considered the bitmaps to calculate CPDFs, PRRs and retransmission count. Henceforth, the term link feature interchanges with either of these other terms, sensed data or data variable.

CPDF is the probability the next packet will be transmitted successfully given a number of consecutive packet successes or failures. CPDFs can give a good visual intuition of link behavior. We wanted to present link behavior between nodes as a single scalar value. To do so, we used Earth Mover’s Distance (EMD) function [62] to compute the retransmission cost. EMD computes the distance between successful packet trace distribution and the failed packet trace distribution with a window size of W=1. While distance is informative, as shown in Table 1, it is not sufficient to measure link quality. Thus, we computed PRR to distill a measure of packet delivery successes and failures within a link. Measuring link behavior and its effect on the network performance, we computed the retransmission count showing the distribution of the number of resumed transmission upon packet failures.

### 4.2. KLSVM Model Implementation

**Figure 7:**
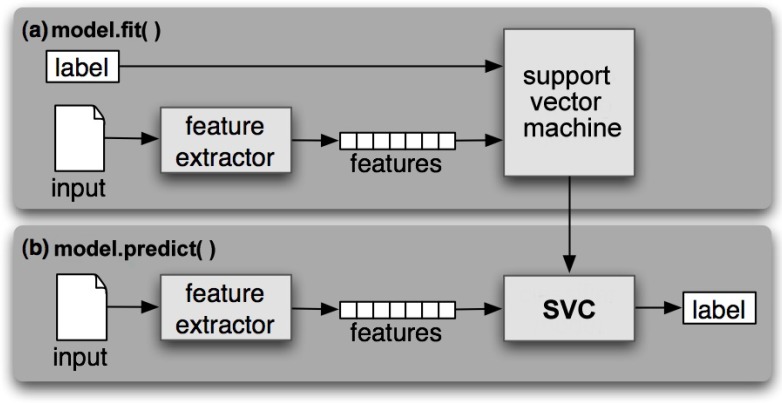
KLSVM’s training and prediction model. The inputs are the pre-processed packet traces.

Using Python’s sklearn library, the support vector classifier (SVC) was computed with C=1 as regularization parameter, and gamma=1 and *decision_function_shape = ’ovr’* as the one-versus-all multi-category classification technique for the linear kernel. For our implementation design (see Figure 7), we used *model.fit()* as an effective method for inputting all the labels (link quality estimates), fitting linear feature data and training the data. The results returned are the optimal values for the parameters. The method *model.predict()* was also used as our prediction model that functioned as an extension to the generic algorithm.

### 4.3. High-Dimensional Multi-Category Classification

KLSVM is a supervised learning multi-category classification technique. It learns a linear kernel function from training data consisting of pairs of pre-processed packet traces, as input features, and link quality estimates, as categorical output. The linear kernel function is used to predict a class label of any valid input feature. Interestingly, within the research field of multi-category classification, there exist two types of existing methods for handling multi-category classification problem that can be distinguished: pairwise classifiers and one-versus-all approach. Whereas the former seeks to solve a series of binary classifications, the latter considers all the classes simultaneously. In our technique, we considered one-versus-all because we required training *X* distinct binary classifiers to separate one class from all others and each binary classifier uses all training samples. For the pairwise approach, there are X(X−1)/2 binary classifiers to be trained with one for each pair of classes. Compared to the one-versus-all approach, the number of classifiers is much larger for the pairwise approach but each one involves only a subsample of the training data and thus is easier to train.

**Figure 8:**
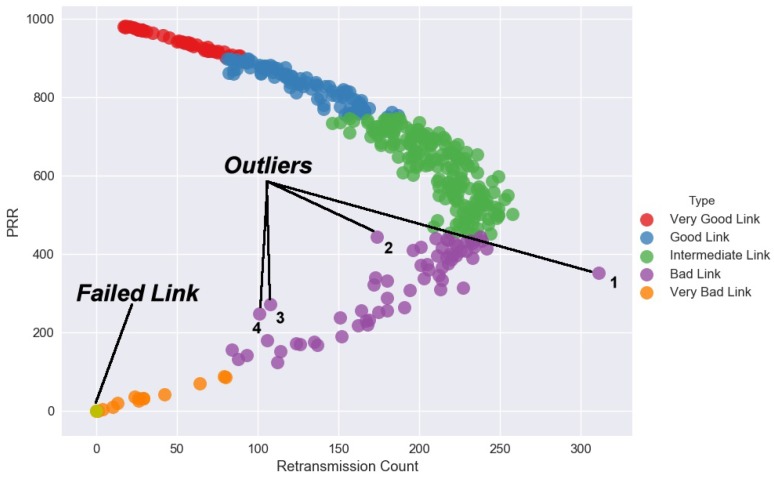
KLSVM Model: A visual intuition of the network behavior.

The remarkable recent development of sense computing, powerful state-of-the-art technology and massive storage technology has allowed scientists to collect data of unprecedented size and complexity. When the dimensionality of the input feature space is too large, things become complicated, for instance, if the raw packet traces were used for training. Another difficulty of high dimensional classification is caused by the existence of many noise features such as outliers that do not contribute to the reduction of classification error. Although individually each extracted feature from the examined packet traces can be estimated accurately, aggregated estimation error over the raw packet traces (if they were to be used as features) can be very large and this could significantly increase the misclassification rate, which places more emphasis on misclassification rates rather than the accuracy of estimated categorical output. Even though in high dimensional classification, the focus is much more about the misclassification rate instead of the accuracy of the estimated parameters. This characteristic makes the LSVM incline to be sensitive to training noisy data. When there exist outliers within their own classes, LSVM classifier inclines to be vigorously affected by such far away points due to the unboundedness of the hinge loss. Figure 8 shows, the directions found by the KLSVM technique puts much more weight on link features that provide large classification power.

Generally, linear prediction methods are likely to perform poorly unless the prediction vector $\hat{y}$ is sparse, that is, the effective number of selected features is small. This is due to, as earlier mentioned, the noise accumulation to a large extent as a high-dimensional problem. Classification technique using all features do not necessarily perform well due to the noise accumulation when estimating a large number of noise features. Dealing with high dimensionality and small sample size mitigates the “curse-of-dimensionality”. The “curse-of-dimensionality” refers to a phenomena that arises when analyzing and organizing the dataset in high-dimensional spaces with very large-dimensional settings that does not occur in low dimensions such as the *3D* physical space.

### 4.4. Spatiotemporal Data Correlation

Since spatiotemporal correlations exist among sensor readings, thus correlation is fundamentally about understanding the direction and strength of the relationship between sensed data variables. Although correlation can be used with hypothesis testing [63], it does a number of other useful roles in this section, as follows:

- Reliability: A strong correlation between link features on a test means that they are consistent with measuring the same behavioral outcome. If all the link features on the test correlate well, then it is a reliable test.
- Validity: When developing a brand new test of intelligence, one could empirically want to demonstrate that the test correlates strongly with an existing measure of intelligence, measuring the same construct as the first intelligence test. A strong correlation between a new test and “Gold Standard” test, such as link quality estimations and their multi-category classification, means they are measuring the same construct and therefore the test is valid.
- Prediction: Experimentally, if link features are strongly correlated such as a drop in packet transmission count and increase in PRR, the next time PRR begins to drop, one can predict that link quality will deteriorate soon. When two data variables are correlated, when one changes, it can be *predicted* what happens with the other.
- Verification: It is for theory verification or testing a theory, if a link feature is causing changes in another link feature, then they are strongly correlated. If this theory is correct, then an outcome *x* should happen. If the link feature turns out to be uncorrelated or are correlated in the wrong direction, then it is concluded that the link feature is certainly not causing changes in others, thus it is noted that the theory is not correct.

Data correlation is examined in our study between three link features, PRR, retransmission count and CPDF (measured in percentage) from the packet traces captured from the 802.15.4 testbed. As earlier seen in Figure 5, the change in one link’s feature, reacted by an equivalent unit change in the other link’s feature directly or indirectly. In other scenarios, variables are said to be uncorrelated in amount when one data variable does not show any movement in another data variable in a specific direction.

**Figure 9:**
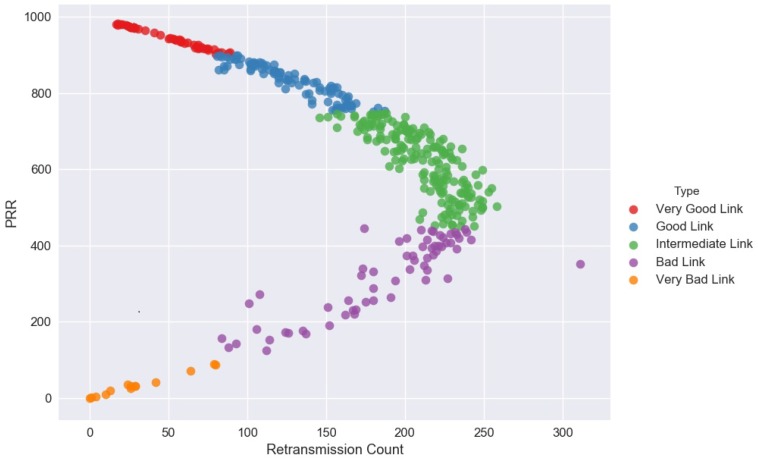
KLSVM: Sensor node observations with PRR greater than 50% have a high degree of negative correlation and a moderate degree of positive correlation for the rest of the observations. These observed links with PRR less than 50% show inconsistent pattern of their retransmission counts, thus the cause of a moderate degree of positive correlation.

Figure 9 shows data correlation can be positive or negative with the two link features moving in the same direction. An increase in one link feature results to a corresponding increase in the other link feature and vice versa. Then, the link features are considered to be positively correlated. On the contrary, when the two link features move in the different direction in such a way that an increase in one link feature will result in the decrease in the other link feature and vice versa, it is empirical said to be negatively correlated.

### 4.5. Detected Anomalies

#### 4.5.1. Outliers

Outliers indicate abnormal sensing conditions and their detection is a critical task in many safety-critical environments that sensors are applied. In our experiment, we observed that four links laid abnormally far away, clearly isolated and inconsistent with the pattern of the other links. Such outcomes could be due to the quality of sensed data that may have been affected by noise, error or inconsistent packet reception. Because outliers are one of the sources that greatly influence the quality of link for transmission, in this section, we provide an overview of the results of detected outliers. Table 2 gives a description of our findings.

KLSVM approach prevented normal sensed data from being classified as outliers and kept the detection rate high and false alarm rate low. The technique identifies outliers from the sensed data measurements but not performed in real-time. The technique pays attention to spatial correlation of neighboring nodes, which makes the results of outliers accurate.

#### 4.5.2. Faulty Link

As earlier seen, Figure 8 shows a detected link failure in the meshed network topology we used for data collection. In a meshed WSN topology, sensor nodes can communicate with numerous nodes within the network, guaranteeing redundancy against connection failures. Connection failures are common in sensing systems because of the inherent unreliability of the shared communication media. Besides, connection disappointments are often caused by hardware failure, software failure, communication failure, aging phenomenon or human error. Since information sent over a fizzled link is lost, the time between a failure and its detection is pivotal for a solid end-to-end communication in the Mesh WSN. Once the link failure is recognized, an effective route is chosen by the routing protocol and communication can proceed. In this way, to increase the unwavering quality of the Mesh WSN, the discovery time for link failures must be kept as low as can be expected under the circumstances. The link failure happened between Node 9 and Node 3 using channel 12. Our investigation reveals that Node 3 temporally lost power at the time Node 9 was establishing its connection. Thus, the detected faulty link was due to a hardware failure.

## 5. Conclusions

Generally, LSVM has the ability to minimize training error and maximize the margin over dataset, whose labels are included as supplemental variables in the optimization problem. While other classification methods focus on conditional probabilities, LSVM targets estimating the decision boundary directly. The LSVM technique performs classification into two classes. However, to perform a high dimensional classification for many classes, the multi-category classification approach using a kernel trick is required.

The Linear kernel trick transforms linearly non-separable data points into higher dimensional feature space where the data points cannot be linearly separated. Kernels are major strength to all SVM systems with small to moderate number of data. Linear kernel is the least common choice, though a powerful feature classifier machine that can be used to design linear functions based on the task at hand.

In this study, we designed a data-driven framework for reliable link classification that models KLSVM to produce stable and consistent results. We present the model based on the real dataset. Our approach modeled and captured the two phenomena—data correlation and high dimensional data classification. Thus, we detected a faulty link and captured four outliers with extremely inconsistent behavior within the network. The technique has a good performance on link quality estimation accuracy.

## Figures and Tables

**Table 1:** Average retransmission cost.

Link Type	Number of Links	Percentage	Average Retransmission Cost ^1^
Very Good Link	49	12.25%	0.1015077
Good Link	93	23.25%	0.309878
Intermediate Link	181	45.25%	0.729711
Bad Link	63	15.75%	1.914515
Very Bad Link	13	3.25%	63. 272 ^2^
Faulty Link	1	0.25%	N/A

^1^ the average retransmission cost in various link quality estimations category; and ^2^ for very bad links it on average costs 63.272 to transmit a packet successfully compared to the other link observations.

**Table 2:** Diagnosis results of the outliers.

Tag ^1^	PRR ^2^	RC ^3^	Chnl ^3^	TL ^3^	Description
1	352	311	16	Node 7 to Node 1	The link exhibits a busty traffic pattern with frequent long packet losses.
2	446	174	12	Node 5 to Node 3	The link exhibits a busty traffic pattern with frequent long packet losses. Notably, after the 780th transmission only 11 packets were delivered.
3	272	108	12	Node 7 to Node 3	The link exhibits a busty traffic pattern with frequent long packet losses. Notably, after the 780th transmission only 5 packets were delivered.
4	248	101	12	Node 6 to Node 3	The link exhibits a busty traffic pattern with frequent long packet losses. Notably, after the 779th transmission only 3 packets were delivered.

^1^ In Figure 8 all the outliers are tagged sequentially. ^2^ The outliers are in the range of “Bad Link” quality estimation. They are ignored because they are not reliable for end-to-end communication. ^3^ Abbreviations as used in the table: RC (Retransmission Count), Chnl (Channel) and TL (Transmission Link).

## Abbreviations

The following abbreviations are used in this manuscript:

SVM	Support Vector Machines
LSVM	Linear SVM
KLSVM	Kernelized Linear Support Vector Machine
SVC	Support Vector Classifier
WSN	Wireless Sensor Network
MEMS	Micro Electromechanical Systems
PRR	Packet Reception Rate
BCM	Beacon-based Measurement
NGMI	New Generation Mobile Internet Research Laboratory
CPDF	Conditional Probability Delivery Function
EMD	Earth Mover’s Distance
